# The effects of values-based digital microinterventions on mental health and valued living, and the role of photography and practice quality: A protocol for a randomized controlled trial

**DOI:** 10.1016/j.conctc.2026.101663

**Published:** 2026-06-29

**Authors:** Pedro Sarrión, Lorena Desdentado, Alejandro Dominguez-Rodriguez, Gerben Westerhof, Ernst Bohlmeijer, Ausiàs Cebolla, Rosa Baños

**Affiliations:** a Polibienestar Research Institute, University of Valencia, Valencia, Spain; b Department of Personality, Evaluation and Psychological Treatment, Faculty of Psychology, University of Valencia, Valencia, Spain; c Department of Psychology, Health and Technology, University of Twente, Enschede, the Netherlands; d CIBERObn Physiopathology of Obesity and Nutrition, Instituto de Salud Carlos III, Madrid, Spain

**Keywords:** Values, Valued living, Values-based interventions, Ecological momentary assessment, Ecological momentary intervention, Mental health

## Abstract

**Background:**

Values are considered central to psychological well-being and mental health, yet the effects of brief interventions targeting valued living remain underexamined. Mobile phone-based delivery may offer a feasible way to implement values-based microinterventions in daily life, while photography may help make values more concrete and accessible. This protocol describes a randomized controlled trial evaluating three values-based microinterventions with and without photography on mental health and values-related outcomes, while also exploring the role of values practice quality as a moderating variable.

**Methods:**

A three-arm, single-blind randomized controlled trial with university students will be conducted. Participants will be randomized to one of three groups: (1) Reading What Matters, a standard written values-based microintervention condition; (2) Capturing What Matters, a photography-supported version of the same microinterventions; or (3) a control group completing only the assessment protocol. Distal outcomes will be assessed at baseline, post-intervention, and two-week follow-up, and will include mental health outcomes, values-related outcomes, and baseline mental imagery ability. Proximal outcomes will be assessed repeatedly through ecological momentary assessments during the intervention period and will include momentary mental health states, momentary valued living processes, and values practice quality.

**Discussion:**

This study will provide initial evidence on the effects of values-based microinterventions on mental health and valued living, the potential contribution of photography, and the role of practice quality in values-focused work.

## Highlights

• The study will evaluate values-based mobile microinterventions in daily life.
• The study will compare the microinterventions with and without photographs.
• Photography will be examined as a strategy to strengthen the effects.
• Values practice quality will be explored as a moderating variable.
• Evidence may support scalable values-based mobile mental health microinterventions.

## 1 Introduction

### 1.1 Values and their role in mental health

Personal values are those aspects that individuals consider important and meaningful in their lives [1]. In research, values have primarily been conceptualized from two distinct theoretical approaches. First, Schwartz's Theory of Basic Human Values (TBHV; [2]) defines values as beliefs about broad desirable objectives that function as guiding principles for decision-making and behavior. This theory organizes 19 *universal* values in a circular structure based on compatible and conflicting motivations: openness to change, self-enhancement, conservation, and self-transcendence. Second, Acceptance and Commitment Therapy (ACT) positions values as a central construct within the Hexaflex model of psychological flexibility [3]. Within this framework, values are conceptualized as freely chosen, verbally constructed life directions that are expressed through ongoing and evolving patterns of action [4].

While Schwartz's theory focuses on the underlying motivational content of values, ACT focuses on formal processes related to how individuals clarify their values and act in accordance with them in daily life, independently of their specific content, which is known as valued living. Recent research indicates that valued living may be a stronger predictor than the motivational content of values for mental health outcomes [5]. Higher levels of valued living have been associated with lower depressive and anxious symptomatology [6] and with higher levels of well-being and meaning in life [7,8]. Moreover, valued living has been identified as a relevant factor in resilience among university students [7]. Together, these findings support the relevance of situating valued living within a broader model of mental health.

The sustainable mental health framework proposed by Bohlmeijer and Westerhof [9] is particularly useful in this regard, as it conceptualizes mental health not only as the absence of psychological distress but also as the presence of well-being and the capacity to adapt to life challenges over time. From this perspective, mental health is shaped by the interplay between psychological resources (e.g., strengths, positive relationships, and meaning) and psychological barriers (e.g., cognitive biases, dysfunctional beliefs, and avoidance) that either support or hinder adaptation. Within this framework, valued living may function as a psychological resource that provides meaning and direction, thereby supporting individuals’ capacity to adapt to life challenges while maintaining psychological well-being over time. As such, valued living may represent a relevant target for psychological interventions aimed at promoting sustainable mental health.

### 1.2 Evidence and open questions in values-based interventions

Values-based interventions (VBIs) are strategies that use personal values as a central component. In the context of mental health, VBIs have mainly focused on promoting valued living by helping individuals clarify what matters to them, identify values-consistent actions, and engage in behavior aligned with those values.

Most research on valued living has been conducted within broader ACT-based interventions that target multiple psychological flexibility processes simultaneously, making it difficult to isolate the specific contribution of values-related processes [10]. However, some studies have isolated valued living as a distinct intervention component. Across these studies, interventions have typically combined reflective exercises to clarify and connect with personally important values, such as psychoeducation and values writing, with more action-oriented exercises aimed at translating values into behavior, such as planning, scheduling, carrying out, and journaling valued actions over time [[11], [12], [13], [14], [15]]. This emerging body of work provides preliminary evidence that VBIs may enhance well-being and reduce psychological distress [14,15], although findings remain mixed [13]. Nevertheless, the evidence base remains limited, and several important questions remain open.

First, many of these studies do not explicitly measure changes in values-related processes, including valued living itself [11,12,14,15], making it difficult to determine whether improvements in mental health are actually driven by changes in valued living. Second, most VBIs have relied on self-guided web-based activities or sessions [[11], [12], [13], [14], [15]]. Such approaches may be less suited to providing immediate, contextually relevant support and to assessing valued living as it unfolds in everyday contexts and fluctuates across moments and days [16]. Therefore, there is a need for research examining whether VBIs delivered in daily life can effectively promote mental health and valued living.

### 1.3 Microinterventions: A promising strategy to study valued living in daily life

A promising way to address these open questions is the use of microinterventions, defined as brief, highly targeted strategies designed to have an immediate impact [17,18]. Because microinterventions focus on specific therapeutic components, they may be particularly useful for examining the effects of valued living strategies in isolation, without the confounding influence of broader multi-component interventions. Moreover, their brief format makes them well suited for delivery in daily life. Initial evidence suggests short-term benefits of microinterventions for mental health outcomes [[19], [20], [21]], supporting their potential as brief strategies that can be integrated into daily life.

Digital mental health interventions, particularly smartphone-based interventions, have received increasing attention in recent years [22], as they provide immediate access to strategies and can be integrated into users' daily lives. In this context, mobile phones represent an ideal channel for delivering microinterventions in individuals’ natural contexts. When microinterventions are delivered at fixed or on-demand times via mobile devices, they are referred to as ecological momentary interventions (EMIs; [23,24]). Mobile phones also enable the collection of ecological momentary assessments (EMAs), which provide real-time measurement of outcomes in daily-life contexts. Meta-analyses have shown their effectiveness in improving mental health [[25], [26], [27]], with small effects on well-being and common symptomatology and moderate effects on emotion regulation [28]. In university students, mobile phone-based interventions have also been shown to reduce symptomatology and risky behaviors, such as alcohol and tobacco use [29,30]. However, mobile phone-based microinterventions specifically targeting valued living as a stand-alone intervention component have received limited attention. This highlights the need to examine values-based microinterventions delivered in daily-life contexts.

### 1.4 Factors that may influence the effects of microinterventions

#### 1.4.1 The role of photography

A key difficulty when targeting values is their abstract and often ambiguous nature [31,32], which may pose challenges for accessing and contacting them. Moreover, this challenge may be even greater in the context of microinterventions, where the time available to engage with personal values is limited. Therefore, strategies that facilitate access to values may be particularly important in this context.

Photography may serve as a promising medium for enhancing values-based microinterventions by transforming abstract values into concrete, personally meaningful visual cues [33]. Through photography-based tasks, such as creating and repeatedly observing participant-produced photographs, individuals may represent their values through specific images, people, places, or moments. These photographs may function as reminders that reactivate associated values when viewed directly, while similar cues encountered in everyday life may also trigger value-related thoughts. For example, if a participant represents the value of “being creative” with a photograph of their guitar, later seeing that instrument at home or elsewhere may serve as a reminder of this value. In this way, photographs may increase the concreteness, salience, and accessibility of personal values, making them more likely to come to mind spontaneously.

Beyond making values more concrete and accessible, observing photographs may also activate broader networks of memories, thoughts, emotions, and mental images associated with personally important values. For instance, viewing the photograph of the guitar may also evoke memories of playing music, feelings of inspiration or enjoyment, thoughts about self-expression, or experiences in which the individual felt engaged or fulfilled. In this way, photographs may provide faster and more direct access to value-related affective states, such as meaning or vitality. In line with this, research has shown that both taking and reviewing photographs can positively influence mood and affect [[34], [35], [36]], facilitate emotion regulation [37], and elicit feelings of meaning in values-focused work [38]. Consistent with broaden-and-build theory [39], these positive and meaningful experiences may broaden individuals’ momentary thought-action repertoires, facilitating additional thoughts, memories, associations, feelings, and value-congruent actions.

Moreover, participant-produced photography may enhance the appeal of values-based microinterventions by increasing personal relevance and a sense of ownership over the intervention content. Because the images are self-generated and connected to the participant's own life, the intervention may feel more personally meaningful and engaging, thereby potentially improving adherence. However, the extent to which participant-produced photography improves the effects of values-based microinterventions remains to be empirically examined.

#### 1.4.2 The role of quality of practice

Another important aspect of VBIs, and of psychological interventions more broadly, is the quality of practice. This refers not only to the content addressed during practice, but also to how effectively that content is implemented [40]. Practice quality has been extensively examined in interventions that rely primarily on meditation, imagination, and visualization, such as those derived from compassion-based traditions [41]. Within this literature, practice quality has often been operationalized through two dimensions: Mental Imagery Ability (MIA) and somatosensory properties [42]. MIA refers to the cognitive processes involved in creating, maintaining, examining, and manipulating mental representations ([43], as cited in Ref. [42]). Somatosensory properties, in turn, refer to the bodily sensations that accompany these experiences.

Similar to compassion-focused approaches, many VBIs designed to foster values clarification involve visualization- and imagination-based strategies. For instance, the “Sweet Spot” exercise invites individuals to recall past meaningful moments and identify the values expressed in those experiences [44]. Other values exercises use future-oriented scenarios, such as “Attending Your Own Funeral”, in which participants imagine their own funeral and reflect on what they would want significant others to say about the life they had lived and the values they had embodied [45]. These exercises suggest that the quality of values practice may depend, at least in part, on the ability to generate and engage with mental imagery.

At the same time, values work may also involve somatosensory and affective components, as LeJeune and Luoma [46] suggest that working with values in therapy often evokes emotional and physical responses. Luoma et al. [47] further describe vitality as a dynamic, vibrant quality that often emerges when individuals deeply connect with their values. Similarly, Barney et al. [48] emphasize that valuing experiences can elicit feelings of vitality and meaningfulness.

Thus, MIA and somatosensory engagement may provide a useful starting point for investigating the quality of values practice. Understanding these internal dimensions is crucial for clarifying the mechanisms through which VBIs achieve their effects. When values practice is supported by stronger mental imagery and somatosensory engagement, individuals may experience a deeper connection with their values and, consequently, greater valued living. This deeper valued living, in turn, is expected to amplify positive effects across dimensions of sustainable mental health. However, few studies have examined the role of practice quality in values-focused work.

### 1.5 Objectives and hypothesis

Based on the literature reviewed above, this study aims to:

(1) Examine the effects of three mobile phone-based microinterventions targeting valued living on mental health and values-related outcomes over one week. Specifically, we aim to investigate effects at three temporal levels: (1.a) proximal effects (immediate changes following each microintervention), (1.b) trajectory effects (changes across days associated with repeated microintervention use), and (1.c) distal effects (changes observed after the intervention period and at the two-week follow-up).
(2) Examine whether photography-supported microinterventions enhance the effects referred to in the first objective compared to non-photography-supported microinterventions.
(3) Explore the moderating role of baseline MIA and momentary quality of values practice on the effects of the microinterventions.

We formulate the following hypotheses:

Hypothesis 1 The three values-based microinterventions will show positive effects on mental health and values-related outcomes across the three temporal levels: (i.e., proximal, trajectory and distal effects).

Hypothesis 2 Photography-supported microinterventions will enhance the effects referred to in Hypothesis 1 compared to non-photography-supported microinterventions across all three temporal levels.

Hypothesis 3 Higher baseline MIA and higher quality of values practice will be associated with stronger microintervention effects referred to in Hypothesis 1 across the three temporal levels.

## 2 Materials and methods

### 2.1 Study design

**Fig. 1:**
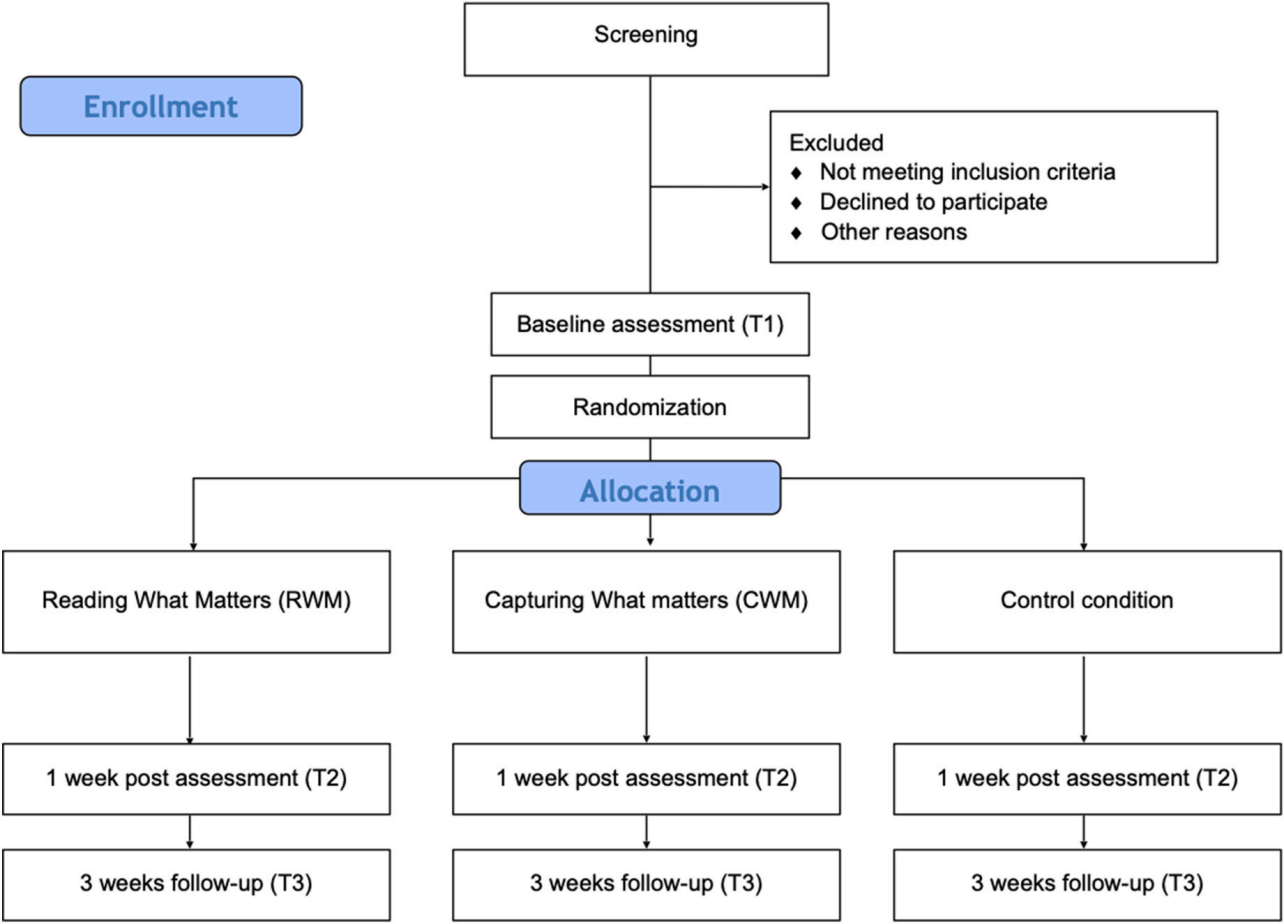
Study design.

A three-arm, single-blind randomized controlled trial with repeated assessments at baseline, post-intervention, and two-week follow-up. EMA surveys will also be administered throughout the intervention period. Eligible participants will be randomly assigned to one of three study conditions in a 1:1:1 ratio: (1) Reading What Matters (RWM), in which participants will complete the values-based microinterventions in a standard written format; (2) Capturing What Matters (CWM), in which participants will complete the values-based microinterventions with the visual support of photography; and (3) control condition, in which participants will not complete any of the microinterventions and will only receive daily notifications to complete the assessment protocol. Fig. 1 shows the study design.

This study will be conducted entirely online, and the microinterventions will be delivered via a study mobile phone application. Participants in all study conditions will be required to download and use the application on their personal smartphones.

### 2.2 Sample size calculation

Currently, few studies have examined the effects of mobile phone-based microinterventions targeting valued living. To date, web interventions targeting valued living have reported effect sizes of *d* = 0.27 for hedonic well-being compared to no treatment [15]. Similarly, Bojanowska et al. [14] found *η*^2^ = 0.09 for hedonic well-being and *η*^2^ = 0.08 for eudaimonic well-being compared to a non-active control group. Therefore, the sample size for this study is determined based on effect sizes observed in prior studies (*f*^*2*^ ranging from 0.19 to 0.31). Specifically, we conducted a power analysis to detect an effect size of *f*^*2*^ = 0.25 for the within-between interaction in a repeated measures ANOVA with three groups (RWM, CWM, control) and three measurement points (baseline, post-intervention/two-week follow-up), with a significance level of 0.05 and statistical power of 0.80. The required sample size for the main analysis was 159 participants in total.

### 2.3 Study population

Recruitment will be conducted at universities in the Netherlands, targeting students pursuing bachelor's, master's, and PhD degrees. Participants will be eligible for inclusion if they are (a) at least 18 years old, (b) have access to a mobile phone and the internet, (c) enrolled as students at a university in the Netherlands, and (d) able to understand and write in English. English will be used as the sole study language to ensure that the same intervention instructions, EMA items, and self-report measures are delivered across participants and conditions. Introducing Dutch or other versions would require formal translation, cultural adaptation, and additional validation to ensure psychometric and conceptual equivalence. It could also introduce subgroup effects that would require additional analyses beyond the scope of the current study.

Exclusion criteria will include (a) being over 45 years old, (b) currently having a diagnosis of a severe mental disorder, such as bipolar disorder, schizophrenia, or borderline personality disorder, (c) taking psychiatric medication, (d) currently receiving psychological or psychiatric treatment for a specific mental disorder, or (e) reporting any suicidal ideation (i.e., scoring above 0 on item 9 of the Patient Health Questionnaire; [49]). Individuals who do not meet the inclusion criteria will be offered alternative resources, such as links to psychological services or opportunities to participate in other lab studies.

### 2.4 Recruitment

The study will be advertised online via LinkedIn, Instagram, and Facebook. In addition, advertisements, including posters and flyers, will be distributed across university campuses. These materials will include a QR code directing interested individuals to the initial study survey and informed consent form. Individuals interested in participating will be required to provide informed consent and complete an initial questionnaire to determine their eligibility based on the inclusion criteria. Additionally, the SONA system, used by the BMS Faculty at the University of Twente, will be used to assign credits to psychology and communication students for their participation.

To account for potential differences associated with recruitment pathways, the source through which participants enter the study will be recorded. Because randomization will occur after enrollment, recruitment sources are expected to be distributed across study conditions. Nevertheless, their distribution will be examined descriptively across study arms to identify any potential imbalance. If meaningful imbalances are observed, sensitivity analyses will be conducted to examine whether recruitment source affects the main findings.

### 2.5 Values-based microinterventions

We developed three values-based microinterventions for this study: 50th birthday party, 3-min wake-up, and friendly reminder. Each microintervention is described in detail below.

#### 2.5.1 Standard microinterventions

Participants assigned to the RWM condition will receive the standard version of the values-based microinterventions described below.

The 50th birthday party aims for helping participants to clarify their central personal values. This microintervention is a 10-min guided visualization in which participants imagine celebrating their 50th birthday surrounded by people they would like to attend. This exercise is a modification of the original ACT strategy *Attending Your Own Funeral* [45]. In the original exercise, individuals are asked to imagine their own funeral, the people that they would love to be surrounded with, and choose three individuals to envision what they might say about them and the life they have lived. In our adapted version, participants will be asked to imagine their 50th birthday party, as this scenario is more accessible and relatable for university students who may find imagining their funeral too distant or complex. This modification also avoids the sensitive topic of death, which could evoke distressing emotions and may be risky in a self-guided intervention without professional support. As in the original exercise, participants select three people and focus on what they would *like* these people to say about them and the life they had lived, rather than what they might probably say. To guide the visualization, participants are prompted with the questions: “What does this person say about how you have lived your life? What does this person say about how you have dedicated your time and energy in recent years? What does this person say about what you have valued as important in your life?” After completing the visualization, participants reflect and write about their experience, answering the following open question: “What words did these people use to describe the person you have become and the life you have chosen to live?” Then, participants will be asked to identify their core values by answering the following question: “After the audio: What things in this world are most important to you above all else?” They will then create a ranked list of 5 core personal values, ordered from most to least important. To help participants articulate meaningful values while avoiding vague or overly simplistic terms, examples will be provided reflecting values as ways of being, acting, or living important for the individual. These examples might include: “For me, taking loving care of my dog is meaningful, or being an ambitious person is something really important for me …” The values written will be stored by the app and will be displayed to them in the following microinterventions.

The 3-min wake-up aims to increase awareness of personal values, identify valued actions, and encourage engagement with them. This microintervention consists of a daily 3-min guided visualization exercise. Before the audio, participants will read their values displayed on the screen, and will be asked to notice what these values evoke in them at that moment. After this, participants will engage with the audio. Firstly, they are encouraged to connect with each value and reflect on why these values are important and meaningful to them. Second, participants are guided to visualize moments during the day in which they could act in line with their values, encouraging them to engage in valued actions and carry this sense of alignment throughout the day.

The friendly reminder encourages participants to become more aware of their personal values throughout the day. This microintervention consists of a text notification. In this strategy, participants will receive a text message prompting them to read and become aware of their personal values. Participants will receive the following message: “Now, take a moment and read your central values”, and their values will be displayed on screen.

#### 2.5.2 Microinterventions with photography

Participants assigned to the CWM condition will receive the same three types of microinterventions as those in the RWM condition, with the addition of a photography component.

The 50th birthday party microintervention will be delivered in the same way as the previous condition. However, after listening to the audio and writing about the experience, participants will be asked to take 5 photos (or select them from their gallery), each representing one of the 5 values they wrote earlier. Then, they will create a folder in their photos app to store these photos, naming the folder as they wish for future use.

The 3-min wake-up will follow the same structure for both groups. However, before the audio, participants will be redirected to the folder where they saved their photos of values. Participants will be asked to notice what these photos evoke in them at that moment. Upon returning to the app, there will be a control question asking if they have looked at the photos.

For the friendly reminder, participants will receive the message: “Now, take a moment, go to your values folder, and look at the photos of your central values”.

### 2.6 Control group

Participants in the control group will not receive any of the microinterventions and will only be assessed daily with the EMA pre-questions, in addition to the assessments at time points 1 (baseline), 2 (post), and 3 (follow-up). After the follow-up, participants in the control condition will receive an email offering them the opportunity to randomly receive one of the two interventions.

### 2.7 Decision rules

**Fig. 2:**
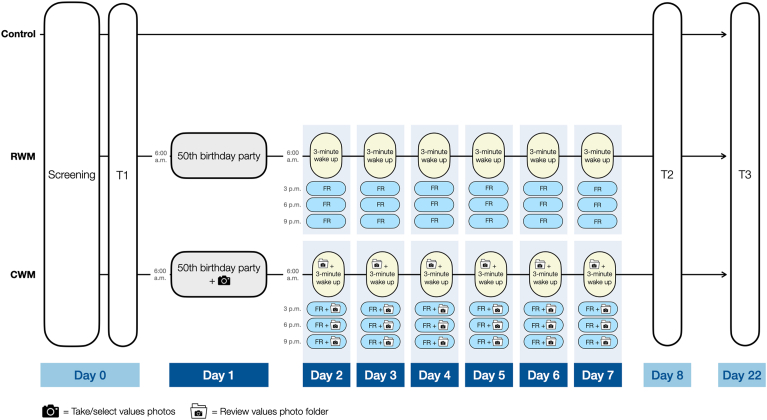
Schedule of microinterventions. Abbreviations: FR: Friendly reminder

Participants will access the microinterventions through push notifications sent via the study mobile phone application, following a predefined time-based sequence (See Fig. 2).

The 50th birthday party microintervention will be sent on the first day after downloading the app, and it will be sent only once with the following message: “It's time to start the journey! Listen to your first visualization”*.* When participants enter the app, this module will be named “Starting your journey!” and will remain available for the next 24 h. Throughout the day, participants will receive two reminders if the strategy has not been completed. The first reminder will be sent at 3:00 p.m. with the message: “Don't forget your first notification! It's really important to us that you complete it”. The second reminder will be sent at 9:00 p.m. with the message: “To stay in the study, please complete today's notification. We'd love to keep you on board!”

The 3-min wake-up microintervention will be sent daily in the morning during the 6 days following the initial microintervention, with the message: “Time for a quick exercise! It will disappear at 12:00 p.m.” When participants enter the app, this module will be named “Before starting your day!” and will be available from 6:00 a.m. to 12:00 p.m. Reminders will be sent at 9:00 a.m. and at 11:00 a.m. with the following messages, respectively: “Don't miss your morning check-in! It takes less than 5 min – Available until 12:00!” and “Don't forget about me! Morning notification disappears in 1 h”

The friendly reminder microintervention will be sent three times per day and will be available from 3:00 p.m. to 5:00 p.m., from 6:00 p.m. to 8:00 p.m., and from 9:00 p.m. to 11:00 p.m., on the same days as the morning notifications They will appear with the message: “Just 1 question”.

### 2.8 Procedure

Interested participants will access it through a link or QR code provided in the advertisements. The study will comprise 4 steps. In Step 1, students will begin by completing an initial web-based survey on Qualtrics. This survey will provide detailed information about the study and include the informed consent form. To minimize response bias, the specific research questions and hypotheses will not be disclosed during participation. The survey will also collect sociodemographic and clinical data, as well as responses to baseline validated questionnaires. Sociodemographic data will include age, gender identity, marital status, education level, nationality, and university enrollment. Clinical data will include information on whether participants are currently receiving psychiatric or psychological treatment for a specific mental disorder and whether they have been professionally diagnosed with a severe mental disorder. Participants who do not meet the eligibility criteria will be excluded due to the emotional sensitivity that the interventions might elicit and the absence of therapist guidance, which could increase the risk of iatrogenic effects. These participants will receive an email thanking them for their interest and informing them that they are not eligible for participation.

Additionally, participants who select any option other than “not at all” on item 9 of the PHQ will receive an email containing links to psychological resources for accessing mental health support. These resources will include https://findahelpline.com/countries/nl and https://www.government.nl/topics/mental-health-services/question-and-answer/help-for-mental-health-problems. These participants will also be excluded from the study.

**Fig. 3:**
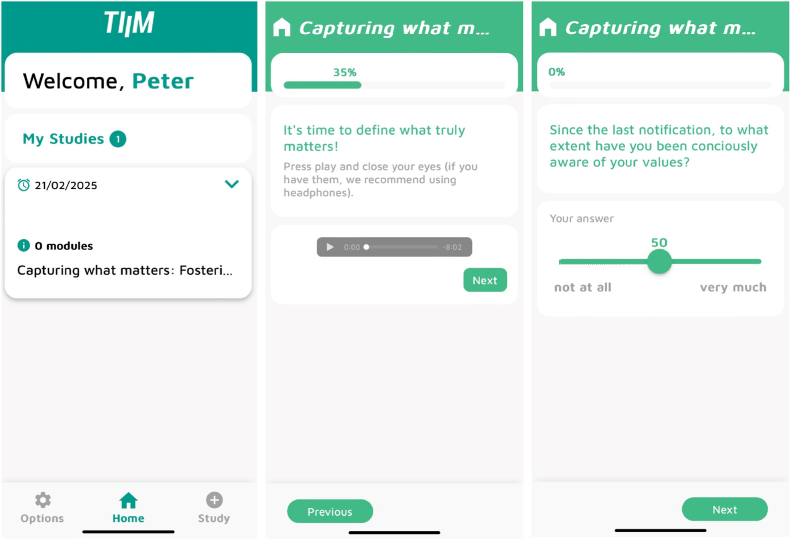
Design of the TIIM mobile-based app intervention.

At the end of the survey, participants will receive instructions for downloading the study mobile phone application, which will also be sent via email for easy reference. The study will be implemented through the TIIM app (Twente Intervention and Interaction Machine; https://www.utwente.nl/en/bmslab/infohub/tiim/; see Fig. 3), a mobile phone application that enables real-time, customized assessments, surveys, and interventions tailored to users’ needs. TIIM will be used to deliver daily notifications, administer EMA questions, provide access to the microinterventions, and collect post-intervention and follow-up assessments when completed through the app. We will not collect passive smartphone-generated data such as GPS location, device usage information, or other automatically generated metadata.

Once participants register in the TIIM app and enroll in the study, they will be randomly assigned in a 1:1:1 ratio to one of three groups: RWM, CWM, or the control group. Randomization will be performed using the Study Randomizer software [50]. After enrollment, participants will receive a schedule outlining the microinterventions to be completed during the study. Reminder emails will be sent to encourage participants to download the app and complete enrollment.

In Step 2, participants will begin receiving daily notifications throughout the 7-day intervention period. These notifications will include the scheduled microinterventions and brief EMA questions administered before and after each exercise.

In Step 3, participants will complete the post-intervention assessment one day after the 7-day intervention period. This assessment will be administered through the TIIM app via a notification that will remain available for 24 h. When participants do not complete the post-assessment within this timeframe, a reminder via email will be sent to the email address provided during the baseline assessment, containing a Qualtrics link to the post-assessment survey. This will allow participants a one-week window to complete the survey, starting from the date of their last uncompleted post-assessment notification.

In Step 4, follow-up data will be collected through a notification will be sent via the TIIM app two weeks after the post-assessment. As with the post-assessment, we will implement the same strategy during the follow-up period, including email reminders, to maximize response rates effectively.

### 2.9 Distal outcomes

Distal outcomes refer to the broader psychological variables expected to change after the intervention period and at the two-week follow-up. These outcomes include both mental health indicators and values-related variables.

#### 2.9.1 Mental health outcomes

Mental health outcomes include:

##### 2.9.1.1 Well-being

The Mental Health Continuum-Short Form will be used (MHC-SF; [51]). This 14-item scale measures three types of well-being: emotional well-being (3 items, e.g., “how often did you feel happy?”), social well-being (5 items, e.g., “how often did you feel that people are basically good”), and psychological well-being (6 items, e.g., “how often did you feel confident to think or express your own ideas and opinions?”). Respondents indicate how often during the past week they experienced each feeling on a 6-point scale, ranging from 0 (never) to 5 (every day). The total score is calculated as a sum of the 14 questions. The total score ranges from 0 to 70, with higher scores indicating higher levels of well-being. The original version of the MHC-SF has good reliability and validity in its English version.

##### 2.9.1.2 Meaning in life

The Meaning in Life Questionnaire will be used (MLQ, [52]). This 10-item scale measures two facets of meaning in life: the presence of meaning (5 items, e.g., “I understand my life's meaning”), and search for meaning (5 items, e.g., “I am looking for something that makes my life feel meaningful”). Items are rated on a 7-point scale, ranging from 1 (absolutely untrue) to 7 (absolutely true). The presence of meaning score is the sum of items 1, 4, 5, 6, and a reversed item 9, while the search for meaning score is the sum of items 2, 3, 7, 8, and 10; both scores range from 5 to 35, with higher scores indicating a greater presence or search for meaning, respectively. The original version of the MLQ has good reliability and validity in its English version.

##### 2.9.1.3 Depression

The Patient Health Questionnaire will be used (PHQ-9; [49]). This 9-item scale measures depressive symptoms (e.g., “Little interest or pleasure in doing things”) during the last week. Items are rated on a 4-point scale, ranging from 0 (not at all) to 3 (nearly every day). The total score is calculated as a sum of the 9 questions. The total score ranges from 0 to 27, with higher scores indicating higher levels of depression. Total sum scores can be interpreted as follows: minimal depression (0 to 4 points), mild depression (5 to 9 points), moderate depression (10 to 14 points), moderately severe depression (15 to 19 points), severe depression (20 to 27 points). The original version of the PHQ-9 has good reliability and validity in its English version.

##### 2.9.1.4 Anxiety

The General Anxiety Disorder Scale-7 will be used (GAD-7; [53]). This 7-item scale measures anxiety symptoms (e.g., “Feeling nervous, anxious or on edge”) during the last week. Items are rated on a 4-point scale ranging from 0 (not at all) to 3 (nearly every day). The total score is calculated as a sum of the 7 questions. The total score ranges from 0 to 21, with higher scores indicating higher levels of anxiety. Total sum scores can be interpreted as follows: no symptoms (0 to 4 points), mild (5 to 9 points), moderate (10 to 14 points), and severe symptoms (15 points or more). The original version of the GAD-7 has good reliability and validity in its English version.

##### 2.9.1.5 Ability to adapt

The Generic Sense of Ability to Adapt Scale will be used (GSAAS; [54]). This 10-item scale measures the ability to readjust after a personally challenging event (e.g., “I can handle setbacks well”). Items are rated on a 5-point scale, ranging from 1 (not at all) to 5 (completely). The total score is calculated as a sum of the 10 items. The total score ranges from 5 to 50, with higher scores indicating better ability to adapt. The original version of the GSAAS has good reliability and validity in its English version.

#### 2.9.2 Values-related outcomes

Values-related outcomes include valued living and values motivational content:

##### 2.9.2.1 Valued living

The Engaged Living Scale will be used (ELS-16; [55]). This 16-item scale measures valued living (VL, items 1-10, e.g., “I have values that give my life more meaning”) and life fulfillment (LF, items 11-16, e.g., “I live the way I always intended to live”) during the last week. Items are rated on a 5 point scale, ranging from 1 (completely disagree) to 5 (completely agree). The total subscales scores are calculated as a sum of the items. Total subscale scores range from 10 to 50 in the VL subscale, and from 6 to 30 in the LF subscale. The total scale score is calculated as a sum of the items. Total scale scores range from 16 to 80, with higher scores indicating higher engaged living. The original version of the ELS-16 has good reliability and validity in its English version.

The Multidimensional Psychological Flexibility Inventory will be used (MPFI; [56]). This 60-item scale measures twelve independent dimensions of the Hexaflex model. For this study, 4 dimensions of the MPFI will be measured with reference to the past week: Contact with values (5 items; e.g., “I was very connected with what is important to me and to my life”); Committed Action (5 items; e.g., “Even in the face of failure I did not stop pursuing what is important to me”; Lack of Contact with Values (5 items; e.g., “Often my priorities and values were left behind in my daily life”; Inaction (5 items; e.g., “Often negative emotions paralyzed me and prevented me from acting”). Items are rated on a 6-point scale, ranging from 1 (Never true) to 6 (Always true). The total subscales scores are calculated as a sum of the 5 items. The total subscales scores range from 5 to 30, with higher scores indicating higher levels of each dimension. The original version of the MPFI has good reliability and validity in its English version.

##### 2.9.2.2 Values motivational content

The Higher-Order-Value Scale-17 will be used (HOVS17; [57]). This 17-item scale measures the 4 higher-order values from the Schwartz's TBHV [2]: openness to change (5 items; e.g., “It is important to her/him to develop her/his own opinions”), self-enhancement (4 items; e.g., “It is important to her/him to show that her/his performance is better compared to the performance of other people”), conservation (3 items; e.g., “It is important to her/him to maintain traditional values and ways of thinking”) and self-transcendence (5 items; e.g., “It is important to her/him to care for nature”). Items are rated on a 6 point scale, ranging from 1 (not like me at all) to 6 (very much like me). The total subscale scores are calculated as a sum of the items. The total subscales scores range from 5 to 25 for openness to change; from 4 to 24 for self-enhancement; from 3 to 18 for conservation; and from 5 to 25 for self transcendence; with higher scores indicating higher importance of each value dimension. The original version of the HOVS17 has good reliability and validity in its English version.

#### 2.9.3 Baseline MIA

MIA will be calculated by combining the scores of imagery controllability and vividness:

##### 2.9.3.1 Controllability

The Test of Visual Imagery Control will be used (TVIC; [[58], [59], [60]]). This 12-item scale measures the ability to control mental images (e.g., “Can you see a car standing in front of a house?”). Items are rated on a 7-point scale, ranging from 0 (cannot see the image at all) to 6 (very easy). The total score is calculated as a sum of the 12 items. The total score ranges from 0 to 72, with higher scores indicating better controllability. The original version of the TVIC has good reliability and validity in its English version.

##### 2.9.3.2 Vividness

The Shortened Questionnaire Upon Mental Imagery (SQMI), a shortened version of Betts’ [61] original Questionnaire Upon Mental Imagery, will be used [62]. This 35-item scale measures vividness of mental imagery on seven sensory modalities: visual, auditory, cutaneous, kinesthetic, olfactory, gustatory and organic (e.g., visual image of “The sun sinking below the horizon”). Items are rated on a 7-point scale, ranging from 1 (No image present at all) to 7 (Perfectly clear). The total score is calculated as a sum of the 35 items. The total score ranges from 35 to 245, with higher scores indicating higher vividness of imagery. The original version of the SQMI has good reliability and validity in its English version. Table 1 provides an overview of the distal outcomes.

**Table 1:** Study variables, questionnaires and assessment time points.

Variable	Questionnaire	S	T1	T2	T3
Well-being	MHC-SF	-	x	x	x
Meaning in life	MLQ	-	x	x	x
Depression	PHQ-9	x	-	x	x
Anxiety	GAD-7	-	x	x	x
Ability to adapt	GSAAS	-	x	x	x
Valued living	ELS-16	-	x	x	x
	MPFI	-	x	x	x
Values content	HOVS17	-	x	x	x
Imagination control	TVIC	-	x	-	-
Imagination vividness	BETT	-	x	-	-

*Note*. S: screening, T1: baseline, T2: post-intervention, T3: two-week follow-up.

### 2.10 Proximal outcomes

Proximal outcomes will be measured before and after completing the microinterventions. The following proximal outcomes will be measured daily using visual analog scales (VAS) ranging from 0 to 100:

#### 2.10.1 Momentary mental health states

Momentary mental health states will include general well-being (“At this moment, what is your level of well-being?”). Following de Vries et al. [63], affective hedonic well-being will be assessed with the item “How happy do you feel right now?”, and cognitive hedonic well-being with the item “At this moment, to what extent are you satisfied with your life?”. Eudaimonic well-being will be assessed using item 14 from the MHC-SF [51], selected because purpose in life is a core component of eudaimonic well-being [64]: “At this moment, to what extent are you feeling that your life has a sense of direction or meaning?”. Depression and anxiety will be assessed using items adapted from the ultra-brief PHQ-4 [65]. Depressive symptoms will be assessed with the items “At this moment, to what extent are you feeling little interest or pleasure in doing things?” and “At this moment, to what extent are you feeling down, depressed, or hopeless?”. Anxiety symptoms will be assessed with the items “At this moment, to what extent are you feeling nervous, anxious, or on edge?” and “At this moment, to what extent are you unable to stop or control worrying?”.

#### 2.10.2 Momentary valued living processes

Because momentary assessment tools for valued living processes remain limited, we developed study-specific EMA items to assess several dimensions of valued living. These included values clarity (“At this moment, how easy is it for you to identify the values that drive your life?”), values awareness (“In the last 24 h, to what extent have you been consciously aware of your values?”), frequency of valued actions (“In the last 24 h, how many of your actions have been aligned with your values?”), awareness during valued actions (“How aware were you, while doing them, that you were acting according to your values?”), and intention to engage in valued actions (“To what extent do you intend to take actions aligned with your values in the following hours?”).

#### 2.10.3 Quality of values practice

The quality of values practice will be measured after the 50th birthday party with the Values Practice Quality Scale (VPQS). Considering that compassion and values-based practices share common elements, such as the use of imagery and bodily sensations, we adapted the Compassion Practice Quality Scale (CPQS) by Navarrete et al. [42] and constructed the VPQS, particularly for this study. The adapted VPQS was based on values literature and incorporates expressions specifically aligned with the values process, ensuring greater relevance and applicability. This 10-item scale measures the values practice quality with two subscales: imagery (items 1,2,3,4,6, and 8), e.g., “During the exercise, I had a lot of difficulty choosing the elements that made up the mental scene) and somatic (items 5,7,9, and 10) e.g., “During the exercise, I noticed feelings of vitality and expansion in my body”. Items are rated on a scale from 0 to 100, indicating the percentage of time their experience aligns with each statement. Higher scores indicate better practice quality (fewer difficulties), except for negative items (items 1, 2, 3, 4, 6, and 8). CPQS has good reliability and validity in its version in Spanish [42]. Reliability and validity of the VPQS remains to be tested. A short 4-item daily questionnaire for assessing the quality of values practice after the 3-min wake-up will be created by selecting the items with the highest factor loadings from the original scale.

Table 2 provides an overview of the proximal outcomes.

**Table 2:** Study variables, EMA items and assessment time points.

Variable	EMA items	50th birthday party		3-min wake-up		Friendly reminder
		Pre	Post	Pre	Post	Pre
Well-being	At this moment, what is your level of well-being?	x	x	x	x	-
	How happy do you feel right now?	x	x	x	x	-
	At this moment, to what extent are you satisfied with your life?	x	x	x	x	-
	At this moment, to what extent are you feeling that your life has a sense of direction or meaning to it?	x	x	x	x	-
Depression	At this moment, to what extent are you feeling little interest or pleasure in doing things?	x	x	x	x	-
	At this moment, to what extent are you feeling down, depressed or hopeless?	x	x	x	x	-
Anxiety	At this moment, to what extent are you feeling nervous, anxious, or on edge?	x	x	x	x	-
	At this moment, to what extent are you not being able to stop or control worrying?	x	x	x	x	-
Values clarity	At this moment, how easy is it for you to identify the values that drive your life?	x	x	x	x	-
Values awareness	In the last 24 h, to what extent have you been consciously aware of your values?/Since the last notification, to what extent have you been aware of your values? (for friendly reminders notifications)	x	-	x	-	x
Frequency of valued actions	In the last 24 h, how many of your actions have been aligned with your values?	x	-	x	-	-
Awareness during valued actions	And how aware were you, while doing them, that you were acting according to your values?	x	-	x	-	-
Intention to engage in valued actions	To what extent do you INTEND to take actions aligned with your values in the following hours?	x	x	x	x	-
Quality of values practice	1. During the practice, I had great difficulty choosing the elements that shaped up the mental scene that I was using to clarify my values.	-	x	-	-	-
	2. During the practice, I had many difficulties constructing the mental scene that I was using to connect with my values.	-	x	-	x	-
	3. During the practice, I had great difficulty holding the mental scene that I was using to clarify my values.	-	x	-	-	-
	4. During the practice, I had a hard time manipulating the mental scene that I was using to connect with my values.	-	x	-	x	-
	5. During the practice, I managed to generate a sense of meaning and significance towards my life.	-	x	-	x	-
	6. During the practice, I had great difficulty seeing clearly and in detail the mental scene that I was using to clarify my values.	-	x	-	-	-
	7. During the practice, I felt that I was completely within the visualization, forgetting that I was in this room.	-	x	-	-	-
	8. During the practice, I self-criticized myself for not being able to clarify my values.	-	x	-	-	-
	9. During the practice, I noticed a sense of vitality or expansion in my body.	-	x	-	x	-
	10. During the practice, I perceived a great connection and sense of closeness with the mental scene that I was using to clarify my values.	-	x	-	-	-

*Note*. Pre = pre-microintervention; Post = post-microintervention.

### 2.11 Statistical analysis

Data will be processed and analyzed with R 4.4.2 [66] or newer and the RStudio interface [67].

First, to test proximal and trajectory effects with and without photography, as well as the moderating role of quality of practice (Objectives 1.a, 1.b, 2, and 3), linear mixed-effects models will be fitted using the *lmerTest* package [68]. Separate models will be fitted for each proximal outcome within each microintervention as the dependent variables. For the 50th birthday party microintervention, participants from the RWM and CWM conditions will be pooled for the pre-post analysis, as the photography component in the CWM condition is introduced only after the post-microintervention EMA. Thus, time (pre- vs. post-microintervention), quality of practice, and their interaction will be entered as fixed-effect predictors. For values awareness, frequency of valued actions and awareness during valued actions, which require time and opportunity to occur or be noticed in daily life, change will be examined using the next available EMA assessment, collected the following day before the 3-min wake-up microintervention. Given that this assessment occurs after the photography task, models including condition will be conducted to examine potential RWM-CWM differences. For the 3-min wake-up microintervention, condition (RWM, CWM), time (pre- vs. post-microintervention), day (from 1 to 6), quality of practice, and their interactions will be entered as fixed-effect predictors. For the friendly reminder microintervention, condition (RWM, CWM, control), time (pre- vs. post-microintervention), day (from 1 to 6), quality of practice, and their interactions will be entered as fixed-effect predictors. Additionally, to examine the combined effect of the 3-min wake-up and friendly reminder microinterventions across the intervention period, linear mixed-effects models will be fitted using the variables assessed in the first EMA assessment of each day as dependent variables. This approach will allow us to examine whether values awareness, frequency of valued actions and awareness during valued actions change across repeated daily engagement with the intervention. Condition (RWM, CWM, control), day (from 1 to 6), and their interaction will be entered as fixed-effect predictors. Participant will be entered as a random-effect predictor in all models. Models will be estimated using restricted maximum likelihood (REML). Under the missing at random (MAR) assumption, all available data will be used, so participants with incomplete EMA data will still contribute to the analyses based on the observations they provide.

Second, to test the distal effects with and without photography on mental health and values-related outcomes (Objectives 1.c and 2), two-way mixed ANOVAs will be conducted with condition (RWM, CWM, control) as the between-subject factor and time (baseline, post-intervention, and two-week follow-up) as the within-subject factor. The inclusion of the MIA as a covariate will be performed if the assumptions for ANCOVA are met (objective 3).

### 2.12 Data management and confidentiality

Given the sensitive nature of the mental health-related information collected in this study, strict confidentiality procedures consistent with the General Data Protection Regulation (GDPR) will be implemented. Participant data will be pseudonymized using unique participant codes, and personal identifying information will be stored separately from research data. Data collected through the TIIM app and online questionnaires will be transferred to and stored on protected institutional servers, with access restricted to authorized researchers. Data handling procedures will comply with institutional ethical requirements and applicable data protection regulations. In the CWM condition, photographs will be used solely as a personal reflective aid on participants’ own devices. The application will not include any function that allows participants to upload or share photographs with the research team. Therefore, no photographic data, including potentially identifiable visual information, will be collected, processed, stored, accessed, or analyzed by the researchers.

## 3 Results

This study is ongoing and data collection is expected to be concluded in July 2025, with plans to publish the study results by November 2025.

## 4 Discussion

This study presents the protocol for a three-arm randomized controlled trial designed to evaluate the effects of three mobile phone-based microinterventions targeting valued living on mental health and values-related outcomes in university students. In addition, the study will examine whether adding a photography component enhances intervention effects and whether quality of practice moderates proximal and trajectory effects.

This study may contribute to the literature at several levels. Theoretically, the study may inform the literature on positive psychological interventions (PPIs). Although values-focused work has been primarily developed within ACT, where values are understood as a key process for promoting psychological flexibility, it also aligns with positive psychology's emphasis on cultivating adaptive psychological resources that support well-being [69]. Following the model of sustainable mental health [9], valued living may also represent a complementary resource-oriented pathway for supporting adaptation, well-being, and mental health over time.

Conceptually, the study approaches valued living as a multidimensional process. This perspective extends traditional approaches that often conceptualize valued living as a unitary construct, allowing for a more fine-grained understanding of which values-related processes are most responsive to intervention and most strongly associated with well-being and mental health.

Regarding the intervention, the study evaluates values-based microinterventions as brief, targeted strategies designed to isolate and examine the specific effects of values work. This is relevant because values-related processes have often been embedded within broader multicomponent interventions, making it difficult to determine their unique contribution to mental health outcomes. The study also compares different values-oriented exercises, including strategies grounded in existing literature (i.e., 50th birthday party) and newly developed theory-informed exercises (i.e., 3-min wake-up and friendly reminder).

In terms of study design, the use of mobile technology enables these microinterventions to be delivered in participants’ daily lives, closer to their real-world experiences. EMIs may increase the accessibility and scalability of values-focused work while enabling the study of valued living as it unfolds in daily life. EMAs, in turn, allow the study to capture immediate changes in mental health and values-related processes, as well as their fluctuations within and across days.

Moreover, the study explores two potential pathways for strengthening the effects of values-focused work: participant-generated photography and quality of practice. Photography may strengthen values-focused work by making values more concrete, salient, and accessible, eliciting meaning-related affective states, and potentially improving adherence. Quality of practice, in turn, represents an initial step toward examining how the way values practices are implemented may influence intervention effects. This focus is particularly relevant because, although practice quality remains underexplored in PPIs, one of its components, mental imagery ability, has already been explored in gratitude and optimism exercises involving imagination-based strategies [70]. Examining these factors may help identify the conditions under which values-based microinterventions are most effective and inform the optimization of future values-based interventions.

The proposed study also presents some limitations that should be considered. First, the English-language requirement may introduce selection bias and limit the generalisability of the findings, as students with lower English proficiency may be less likely to participate. Making the study materials available in both English and Dutch may broaden accessibility and enable future cross-cultural investigations. Moreover, participants may differ in motivation, expectations, or engagement depending on the pathway through which they enter the study, particularly when participation is linked to course credit. Although participants will be randomized and recruitment pathways will be recorded and examined across study arms, residual differences related to recruitment context may still influence adherence or outcome responses. Attrition is also expected in longitudinal experimental designs, especially in mobile phone-based interventions [[71], [72], [73]]. Difficulties in completing the tasks assigned during the intervention or a lack of understanding of the intervention rationale may increase this risk. To minimize the likelihood of drop-out, a predefined schedule with the study notifications will be sent to participants before the intervention period begins. A further limitation concerns the use of study-specific EMA items. Although these items were designed to assess theoretically relevant momentary constructs, their reliability and validity is yet to be established, and results involving these measures should be interpreted cautiously. Additionally, research has shown that internet-based interventions may have negative effects in some cases [74], including the worsening of existing symptoms or the emergence of new symptoms not directly addressed by the intervention. Values are also a sensitive topic and may evoke a range of emotional reactions. To provide support, a contact number and email address will be available throughout the study, particularly at the beginning of the intervention. This support will be available for both technical difficulties related to the app and emotional issues arising from the intervention. From the start of the study until the follow-up assessment, participants will be able to contact the research team by phone, text message, or email if they experience any of these difficulties. Previous research has shown that support in internet-based interventions is positively associated with treatment outcomes [75].

Overall, this protocol outlines a theory-grounded and ecologically informed approach to examining valued living in daily life. By focusing on values-based microinterventions, proximal processes, and factors that may shape intervention effects, the study may offer useful directions for future research and applied work in mental health promotion.

## Consent to participate

All participants must provide written informed consent prior to participating.

## Consent to publish

All participants must provide written informed consent for publication.

## Ethics approval

The study received ethical approval from the Institutional Review Board of the Faculty of Behavioural, Management, and Social Sciences at the University of Twente (ethical approval number: 201467) and has been pre-registered at the Open Science Framework (OSF; https://osf.io/dztnk). The study will be conducted in accordance with CONSORT 2010 76, CONSORT-EHEALTH 77, SPIRIT guidelines 78 and the principles of the Declaration of Helsinki.

## Funding statement

This work was supported by Grant Prometeo Programme for Research Groups of Excellence (CIPROM/2021/041—Project “IMPULSA”) (Conselleria de Educación, Cultura, Universidad y Empleo; Generalitat Valenciana). P.S. was supported by the PhD scholarship “Atracció de Talent” (UV-INV-PREDOC22-2232336) from the Research Grant Program of University of Valencia.

## CRediT authorship contribution statement

**Pedro Sarrión:** Conceptualization, Investigation, Methodology, Validation, Visualization, Writing – original draft, Writing – review & editing. **Lorena Desdentado:** Formal analysis, Investigation, Methodology, Visualization, Writing – review & editing. **Alejandro Dominguez-Rodriguez:** Conceptualization, Investigation, Methodology, Supervision, Validation, Writing – review & editing. **Gerben Westerhof:** Conceptualization, Investigation, Methodology, Resources, Supervision, Validation, Writing – review & editing. **Ernst Bohlmeijer:** Conceptualization, Investigation, Methodology, Supervision, Validation, Writing – review & editing. **Ausiàs Cebolla:** Conceptualization, Investigation, Methodology, Supervision, Validation, Writing – review & editing. **Rosa Baños:** Conceptualization, Funding acquisition, Investigation, Methodology, Project administration, Resources, Supervision, Validation, Writing – review & editing.

## Declaration of competing interest

The authors declare that they have no known competing financial interests or personal relationships that could have appeared to influence the work reported in this paper.

## Data Availability

No data was used for the research described in the article.
